# The fascination of values: making use of ethics in public health

**DOI:** 10.1093/pubmed/fdaf094

**Published:** 2025-12-10

**Authors:** Andreu Segura, Angel Puyol, Valle Coronado, Francisco J Garcia-León, Eduard Satué de Velasco

**Affiliations:** Grupo de trabajo de Etica de SESPAS: Sociedad Española de Salud Pública y Administración Sanitaria (SESPAS Ethics Working Group: Spanish Society of Public Health and Health Administration), Dr. Ribalta, 5 c14 08960 Sant Just Desvern, Barcelona, Spain; Departamento de Filosofia Facultat de lletres Universitat Autònoma de Barcelona C/Fortuna s/n 08193 Cerdanyola del Vallès, Spain; Medicina Familiar Universidad Francisco de Vitoria Ctra. Pozuelo a Majadahonda, Km 1.800 28223 Pozuelo de Alarcon, Spain; Departamento de Metafísica Facultad de Filosofía Universidad de Sevilla Camilo José Cela s/n 41018 Sevilla, Spain; Community Pharmacist San Francisco, 3050710 Maella (Zaragoza), Spain

**Keywords:** ethics, public health

## Abstract

Public health measures intended to address collective health problems can be harmful or inconvenient for certain population groups. This hinders the effective enforcement of these measures, even when they are legal norms. In such cases, appealing to culturally accepted moral values can be an effective persuasion strategy to increase public support. This is an example of the usefulness of ethics for public health.

## Introduction

Ethics plays a role in social, academic, and professional debates in public health, particularly when conflicts of interest are revealed, such as those between collective and private rights, which expose moral discrepancies. Such debates usually involve deontological and consequentialist ethical theories, and references to philosophers such as Kant and Bentham are common, even though neither considered the ethical challenges of public health.

The ethical dilemma between principalism and consequentialism, as seen in various forms of utilitarianism, often creates more problems than it solves when it comes to public health. A more practical approach would be to adopt a perspective based on cooperation and consensus to find optimal solutions rather than competing with one another.

The ethical dilemma posed by potential confinement could be approached in two ways. One approach is analogous to the Trolley Problem,^1^ which inevitably creates winners and losers. The other approach is a win-win strategy that includes the most vulnerable people, who would suffer the worst consequences of a collective health problem.^2^

Public health can be viewed as an arena of confrontation between opposing interests, where only one moral truth exists, or as a scenario of mutual care. In the latter, it is necessary to agree on the most reasonable approach, rather than the most rational one. This approach is more in line with the uncertain world we live in.

When faced with a public health problem, health and political authorities often exhibit a mixture of epistemological arrogance and moral paternalism. Experts and political authorities easily cast themselves as the community’s saviors or protective parents, even if they intend to generate trust.

However, the result is often the opposite. People distrust science because they believe it has the answer to everything and politicians because they hide their fears and doubts in the face of uncertainty. In the context of public health, an attitude of recognizing that we don’t know everything and that the proposed measures might not be as effective as desired will likely generate more public confidence than an arrogant attitude.

In these situations, appealing to seductive values and virtues could increase the trust essential to optimizing the response to health authorities’ recommendations and standards. This could complement the stewardship model adopted by the Nuffield Trust.^3^

Therefore, this contribution considers using moral values as a persuasive element to convince the population to adopt public health measures that, although less appealing to public opinion at first, better respond to the population’s needs. Of course, this does not preclude the promulgation of mandatory standards if necessary.

### Justification

Health authorities have different levels of legitimacy when implementing public health measures. A first level of legitimacy comes from a legal mandate, which increases when transparent and well-defined procedures are implemented. However, these measures are more legitimate, and their effects are more readily accepted, when the moral values of the population are also taken into account. In a pluralistic society, these values are often conflicting.

The effectiveness of coercive rules depends on the authorities’ material capacity to enforce them. This requires sufficient resources from state security forces and sufficient discipline from the population, which depends on confidence in the competent authority.

Trust can help generate this judgment, as perhaps happened at the beginning of Neolithic urbanization with the implementation of sanitation activities such as access to drinking water, food storage and preservation, waste disposal, and burial of corpses. These were essential measures for the first cities to survive for centuries, as was the case in Çatalhöyük^4^ and Jericho.^5^

Trust and social approval remain as important today as ever.^6^ They require the cultivation of attitudes, behaviors, and customs that foster social coexistence. These attitudes, behaviors, and customs deserve to be valued as good, just as those that hinder social coexistence deserve to be qualified as bad. In both cases, these are examples of moral judgments.

Interestingly, the Latin etymology of ‘moral’ is ‘mor-mores,’ which means ‘custom,’ as does the Greek etymology of ‘ethics,’ ‘ethos.’ This etymological convergence even anecdotally links the origins of ethics and public health.

### Strategic elements for implementation

To materialize this initiative—using ethics as a persuasive element—the following questions must be answered: What, how, and where?

#### What?

The ‘what’ refers to the ethical criteria—values and virtues—to be applied. These are the propositions that are most attractive as elements of popular persuasion. In theory, ancestral dispositions should work. Among these, Sauer highlights cooperability,^7^ though in today’s individualistic societies, adopting the behavior of freeloaders is quite easy. Sauer himself contrasts this behavior with punishment as a beneficial—and therefore morally positive—way to neutralize freeloaders.

In any case, it is imperative to discover or rediscover the values and virtues that generate greater social responsibility and cooperation for the common good among individuals.

#### How?

Although appealing to the moral values and virtues identified as the most persuasive is an option for social marketing, it is vulnerable to propaganda manipulation. Therefore, the moral debate should be framed in a context that facilitates transparency, criticism of single-mindedness, and the demand for systematic accountability.

To this end, the greatest possible citizen participation must be achieved, and one way to do so is to promote public deliberation. This deliberation would be based on values that we all formally want to defend, such as justice, freedom, and beneficence in health. However, it would take into account equity and not discriminate against the interests of the least favored.

Deliberation is not negotiation; it is confronting various interests from a moral perspective. In other words, what benefits one party must benefit or at least not harm the community as a whole. From the point of view of Kantian ethics, moral judgment should counteract the sum of ‘selfish’ preferences that predominate in mere negotiations between particular interests.

In ancient Greek democracy, freedom was understood to be not only the autonomy to decide what is in one’s own interest, but also participation in public affairs.^8^ In the context of public health, this implies that citizens participate in decisions through a deliberative process.

An adequate response to public health problems requires ethical values such as trust, transparent information, accessible information, and citizen participation in decision-making to dialogue, truthfulness, honesty, solidarity, responsibility, respect for people’s dignity, equity, and accountability are essential.

Those responsible for public health must consider the importance of providing the population with truthful and sufficient information on the effectiveness of the proposed measures, as well as their potential harms and benefits.^9^ This is evident in controversial decisions, such as confining the population during a public health crisis or mandating vaccinations.

Transparency is linked to trust. However, striking a balance between these values in public health is difficult since clear information is necessary to generate trust. This is evident when communicating the risks associated with recommended vaccines, for example. In any case, transparency, honesty, accountability, and citizen participation in decision-making can promote trust and improve the implementation of measures.^10^

Public health is particularly interested in reducing social inequalities because they are the origin of health problems. People with fewer economic resources, a lower level of education, or who live in places with poor sanitation are more likely to develop diseases, as seen with the spread of the novel coronavirus.^11^ Addressing social inequalities respects and defends the equity and dignity of individuals.

Tackling public health problems requires institutional responsibility to promote healthy habits and provide the means to do so. However, individuals also have a responsibility to maintain their own health. The value of responsibility is linked to solidarity, or sacrificing one’s own interests for the benefit of the community. Public health is a community matter that permanently requires recourse to values of social commitment, among which solidarity stands out.

In pluralistic and democratic societies, where different ethical values come into tension, public health decision-making requires more than the participation of those involved, whether in the form of consultations or citizen panels. Rather, a process of public or citizen deliberation is necessary to make informed decisions that consider the common good.^12^ Thus, whenever possible, decisions in this area should be based on deliberative processes,^13^ including those transmitted through social marketing.

This deliberative public health process far transcends national societies, whether pluralistic or democratic. The recent pandemic has reminded us that many public health problems exist beyond epidemics and include water management and climate change. In this sense, deliberation and the search for consensus involve distant cultural traditions that often lack a democratic basis. What unites us is mutual interest: ‘Better to agree because the alternative is worse’.

Therefore, international treaties serve as a minimum reference. Public health on a global scale is a specific example of international law, which all involved parties understand to be essential. These codes can be imposed or agreed upon by the parties involved. In any case, it is better to share a code than not to have one, since existing codes can be improved.

International agencies, including the WHO, must ensure that these codes, such as the agreement recently approved by the World Health Assembly, adhere to Cicero’s maxim, ‘Salus populi suprema lex est’, meaning ‘the common good is the supreme law,’ since, in our globalized world, the people to whom this law appeals are inevitably everyone’s people.

#### Where? In what spheres?

The dimensions of public health as a social institution are not limited to governmental organizations with political responsibilities and coercive capacities.^14^ They also include spaces within the healthcare system, such as medical specialties or preventive nursing.

In some systems, they are even part of primary and community healthcare. Fields of research and teaching, which may or may not be linked to the governmental dimension, should also be considered. In Spain, for example, public health research is carried out in public administration institutions, such as the *Instituto de Salud Carlos III* (IS CIII) and the *Consejo Superior de Investigaciones Científicas* (CSIC); in universities (both public and private); and in entities attached to the healthcare system. Many of these entities are integrated into the *Ciberesp* (Center for Networked Biomedical Research in Epidemiology and Public Health).

While the administrative or governmental dimension of public health holds the most executive responsibility, the involvement of health care, teaching, and research elements related to the protection and promotion of collective health is fundamental, precisely because of their independence from executive power, at least formally.

Identifying culturally respected values and virtues can help convince the population, including disadvantaged social groups, of the need for controversial public health interventions.

## Data Availability

We hereby declare that no personal or statistical data were used in the preparation of this manuscript. All information and data referenced in the article are derived exclusively from publicly available bibliographic sources. No individual-level data or sensitive information were collected, analyzed, or reported. As such, ethical approval and informed consent were not required for this study.

## Notes

**Andreu Segura,** MD PhD

**Angel Puyol**, PhD Profesor of Philosophy

**Valle Coronado**, MD PhD

**Francisco J. Garcia-León**, MD PhD

**Eduard Satué de Velasco**, Pharmacist PhD
